# Supplementary material for: A Comprehensive QSAR Study on Antileishmanial and Antitrypanosomal Cinnamate Ester Analogues
Source: Molecules. 2019 Nov 28;24(23):4358. doi: 10.3390/molecules24234358 (PMC6930487; doi:10.3390/molecules24234358)
Supplement: Supplementary file 1 [file molecules-24-04358-s001.pdf]

A Comprehensive QSAR Study on Antileishmanial and Antitrypanosomal Cinnamate Ester Analogues

Freddy A. Bernal [1] and Thomas J. Schmidt [1,*]

[1] Institut für Pharmazeutische Biologie und Phytochemie (IPBP); Westfälische Wilhelms Universität – Münster; PharmaCampus - Corrensstraße 48, D-48149, Münster, Germany; f.bernal@uni-muenster.de
* Correspondence: thomschm@uni-muenster.de; Tel.: +49 251 83 33378.

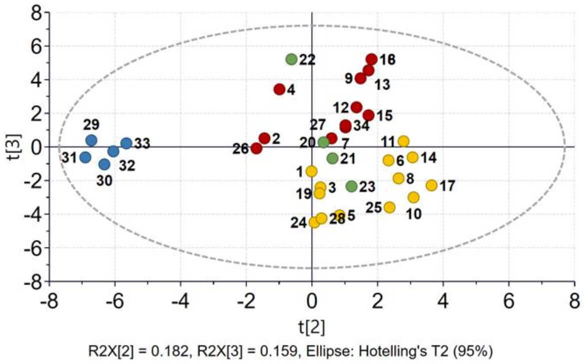

Figure S1. PC2–PC3 score plot of the principal component analysis (PCA) using MACCS fingerprints.

Table S1. Antiparasitic activity of the cinnamate ester analogues [1].

Compound	pIC_{50}_Ld	pIC_{50}_Tbr	Compound	pIC_{50}_Ld	pIC_{50}_Tbr
**1**	3.75	3.97	**18**	6.69	6.90
**2**	4.16	4.15	**19**	4.66	4.59
**3**	4.15	4.21	**20**	5.92	3.70
**4**	4.53	4.25	**21**	5.78	4.10
**5**	4.49	4.42	**22**	6.38	3.85
**6**	3.79	3.73	**23**	5.88	3.95
**7**	3.99	4.14	**24**	4.72	4.24
**8**	4.34	4.30	**25**	4.81	4.50
**9**	4.69	4.64	**26**	3.67	3.91
**10**	4.67	4.34	**27**	4.11	3.80
**11**	5.06	5.60	**28**	4.74	4.39
**12**	5.25	5.50	**29**	4.05	4.12
**13**	5.82	6.10	**30**	4.30	4.00
**14**	5.68	6.00	**31**	3.29	3.62
**15**	4.62	5.86	**32**	4.17	3.68
**16**	6.19	6.45	**33**	3.32	3.62
**17**	5.58	5.92	**34**	3.60	3.99

Table S2. Validation parameters.

Parameter	Mathematical definition	Reference
1	$R^2 = 1 - \frac{\sum(Y_{obs} - Y_{pred})^2}{\sum(Y_{obs} - \overline{Y_{obs}})^2}$	[2]
2	$F = \frac{\frac{\sum(Y_{pred} - \overline{Y_{obs}})^2}{n}}{\frac{\sum(Y_{obs} - Y_{pred})^2}{N-n-1}}$	[2]
3	$Q^2 = 1 - \frac{\sum(Y_{obs} - Y_{pred(LOO)})^2}{\sum(Y_{obs} - \overline{Y_{obs}})^2}$	[2]
4	$R_p^2 = \frac{\sum(Y_{obs} - \overline{Y_{obs}})(Y_{pred} - \overline{Y_{pred}})}{\sqrt{\{[\sum(Y_{obs} - \overline{Y_{obs}})][\sum(Y_{pred} - \overline{Y_{pred}})]\}^2}}$	[3]
5	$R_0^2 = 1 - \frac{\sum(Y_{pred} - kY_{pred})^2}{\sum(Y_{pred} - \overline{Y_{pred}})^2}$	[3]
6	$R_0'^2 = 1 - \frac{\sum(Y_{obs} - k'Y_{obs})^2}{\sum(Y_{obs} - \overline{Y_{obs}})^2}$	[3]
7*	$R_m^2 = R_p^2 \times \left(1 - \sqrt{(R_p^2 - R_0^2)}\right)$	[4–6]
8*	$R_m'^2 = R_p^2 \times \left(1 - \sqrt{(R_p^2 - R_0'^2)}\right)$	[4–6]
9	$\overline{R_m^2} = \frac{R_m^2 + R_m'^2}{2}$	[4–6]
10	$\Delta R_m^2 = \lvert R_m^2 - R_m'^2 \rvert$	[4–6]
11	$Q_{F1}^2 = 1 - \frac{\sum(Y_{obs(test)} - Y_{pred(test)})^2}{\sum(Y_{obs(test)} - \overline{Y_{train}})^2}$	[2]
12	$Q^2{}_{F2} = 1 - \frac{\sum(Y_{obs(test)} - Y_{pred(test)})^2}{\sum(Y_{obs(test)} - \overline{Y_{test}})^2}$	[7]
13	$Q^2{}_{F3} = 1 - \frac{\left[\sum(Y_{obs(test)} - Y_{pred(test)})^2\right]/N_{test}}{\left[\sum(Y_{obs(test)} - \overline{Y_{train}})^2\right]/N_{train}}$	[8]
14	$CCC = \frac{2\sum(Y_{obs} - \overline{Y_{obs}})(Y_{pred} - \overline{Y_{pred}})}{\sum(Y_{obs} - \overline{Y_{obs}})^2 + \sum(Y_{pred} - \overline{Y_{pred}})^2 + N_{test}(\overline{Y_{obs}} - \overline{Y_{pred}})^2}$	[9,10]
15	$MAE = \frac{\sum\lvert Y_{obs(test)} - Y_{pred(test)}\rvert}{N_{test}}$	[11]
5a	$k = \frac{\sum Y_{obs} Y_{pred}}{\sum Y_{pred}^2}$	[3]
6a	$k' = \frac{\sum Y_{obs} Y_{pred}}{\sum Y_{obs}^2}$	[3]
7a and 8a	$Scaled\ Y_i = \frac{Y_i - Y_{min}}{Y_{max} - Y_{min}}$	[6]

* Calculations of R_0^2 and $R_0'^2$ to be used here must be scaled (Scaled Yi)

Table S3. Statistical validation of the models predicting antileishmanial activity.

Model	R^2	Q^2	F	R_0^2	$R_0'^2$	R_p^2	Q_{F1}^2	Q^2_{F2}	Q^2_{F3}	CCC	MAE	σ_{MAE}	$\overline{R_m^2}$	ΔR_m^2
M1-1	0.927	0.906	92.82	0.753	0.753	0.768	0.679	0.679	0.786	0.839	0.355	0.257	0.515	0.235
M1-2	0.921	0.900	85.74	0.671	0.586	0.675	0.503	0.503	0.668	0.733	0.446	0.313	0.482	0.255
M1-3	0.909	0.888	72.87	0.846	0.801	0.848	0.718	0.718	0.812	0.844	0.298	0.289	0.701	0.135
M1-4	0.905	0.886	69.78	0.699	0.560	0.699	0.654	0.654	0.769	0.794	0.363	0.275	0.532	0.230
M1-5	0.911	0.884	74.86	0.738	0.747	0.759	0.724	0.724	0.816	0.863	0.308	0.268	0.680	0.067
M2-1	0.896	0.868	45.16	0.968	0.970	0.971	0.965	0.965	0.977	0.983	0.134	0.047	0.905	0.027
M2-2	0.893	0.855	43.59	0.245	0.281	0.397	0.227	0.227	0.484	0.628	0.414	0.556	0.268	0.137
M2-3	0.891	0.841	43.07	0.810	0.825	0.827	0.810	0.810	0.873	0.909	0.288	0.171	0.681	0.148
M2-4	0.883	0.830	39.81	0.939	0.946	0.947	0.939	0.939	0.959	0.971	0.159	0.106	0.917	0.043
M2-5	0.897	0.819	45.53	0.702	0.423	0.719	0.701	0.701	0.800	0.803	0.318	0.284	0.628	0.092
M3-1	0.892	0.817	32.93	0.604	0.480	0.612	0.597	0.597	0.731	0.774	0.397	0.288	0.438	0.271
M3-2	0.881	0.814	29.64	0.935	0.940	0.940	0.927	0.927	0.951	0.964	0.172	0.118	0.890	0.050
M3-3	0.862	0.813	24.88	0.861	0.753	0.903	0.782	0.782	0.855	0.862	0.244	0.273	0.837	0.078
M3-4	0.880	0.805	29.46	0.653	0.428	0.654	0.627	0.627	0.751	0.766	0.337	0.339	0.468	0.260
M3-5	0.855	0.786	23.65	0.055	–0.402	0.194	–0.224	–0.224	0.182	0.349	0.555	0.670	0.107	0.027
M4-1	0.933	0.918	101.97	–1.495	0.500	0.642	–1.514	–1.525	–1.161	0.585	0.739	1.181	0.426	0.296
M4-2	0.933	0.918	101.97	–1.495	0.500	0.642	–1.514	–1.525	–1.161	0.585	0.739	1.181	0.426	0.296
M4-3	0.929	0.911	96.24	0.710	0.676	0.719	0.688	0.686	0.731	0.832	0.397	0.265	0.619	0.159
M4-4	0.929	0.911	96.24	0.710	0.676	0.719	0.688	0.686	0.731	0.832	0.397	0.265	0.619	0.159
M4-5	0.923	0.910	87.83	0.811	0.800	0.816	0.808	0.807	0.835	0.902	0.309	0.212	0.634	0.177
M5-1	0.933	0.906	72.94	0.918	0.895	0.923	0.918	0.918	0.929	0.954	0.196	0.149	0.774	0.085
M5-2	0.927	0.902	66.74	0.912	0.872	0.932	0.904	0.904	0.918	0.942	0.201	0.175	0.814	0.070
M5-3	0.927	0.902	66.74	0.912	0.872	0.932	0.904	0.904	0.918	0.942	0.201	0.175	0.814	0.070
M5-4	0.931	0.901	70.92	0.849	0.789	0.855	0.841	0.841	0.864	0.906	0.272	0.207	0.718	0.133
M5-5	0.927	0.900	66.56	0.899	0.862	0.909	0.892	0.891	0.907	0.937	0.227	0.167	0.770	0.094
M6-1	0.950	0.924	76.59	0.833	0.826	0.837	0.833	0.832	0.857	0.915	0.264	0.233	0.726	0.139

**Model**	R^2	Q^2	F	R_0^2	$R_0'^2$	R_p^2	Q_{F1}^2	${Q^2}_{F2}$	${Q^2}_{F3}$	CCC	MAE	σ_{MAE}	$\overline{R_m^2}$	ΔR_m^2
M6-2	0.945	0.922	68.17	0.934	0.915	0.941	0.934	0.933	0.943	0.962	0.169	0.144	0.826	0.062
M6-3	0.951	0.920	77.36	0.846	0.838	0.849	0.845	0.845	0.867	0.921	0.254	0.225	0.737	0.131
M6-4	0.934	0.910	56.98	0.902	0.864	0.914	0.901	0.901	0.915	0.943	0.228	0.141	0.744	0.099
M6-5	0.938	0.908	60.82	0.920	0.897	0.927	0.920	0.920	0.932	0.955	0.201	0.134	0.767	0.085
M7-1	0.922	0.899	86.59	0.832	0.685	0.875	0.816	0.746	0.782	0.837	0.369	0.190	0.699	0.125
M7-2	0.899	0.866	65.58	0.735	0.354	0.802	0.700	0.586	0.644	0.722	0.463	0.260	0.590	0.190
M7-3	0.899	0.865	65.41	0.734	0.488	0.757	0.654	0.522	0.590	0.708	0.459	0.348	0.637	0.181
M7-4	0.872	0.843	49.94	0.730	0.521	0.744	0.650	0.517	0.585	0.712	0.473	0.331	0.625	0.187
M7-5	0.873	0.842	50.25	0.752	0.549	0.773	0.687	0.568	0.629	0.737	0.475	0.263	0.532	0.224
M8-1	0.935	0.915	75.46	0.776	0.487	0.845	0.799	0.723	0.762	0.808	0.385	0.199	0.731	0.126
M8-2	0.922	0.900	62.21	0.753	0.682	0.753	0.592	0.437	0.516	0.712	0.534	0.315	0.593	0.197
M8-3	0.917	0.887	58.11	0.766	0.449	0.837	0.746	0.649	0.699	0.762	0.438	0.214	0.632	0.163
M8-4	0.921	0.883	61.23	0.760	0.546	0.786	0.714	0.605	0.660	0.753	0.454	0.251	0.558	0.209
M8-5	0.915	0.880	56.65	0.673	0.454	0.675	0.661	0.532	0.598	0.718	0.467	0.323	0.493	0.248
M9-1	0.961	0.941	98.14	0.786	0.714	0.787	0.830	0.765	0.798	0.864	0.362	0.167	0.644	0.171
M9-2	0.955	0.934	84.24	0.832	0.642	0.904	0.851	0.794	0.823	0.860	0.299	0.232	0.867	0.073
M9-3	0.948	0.926	72.56	0.823	0.689	0.854	0.807	0.734	0.771	0.833	0.382	0.184	0.659	0.148
M9-4	0.946	0.923	70.37	0.825	0.713	0.845	0.805	0.730	0.768	0.835	0.394	0.163	0.678	0.143
M9-5	0.943	0.920	66.52	0.824	0.731	0.836	0.811	0.739	0.776	0.844	0.375	0.191	0.632	0.164
M10-1	0.972	0.958	111.28	0.903	0.860	0.920	0.892	0.850	0.871	0.911	0.267	0.177	0.861	0.065
M10-2	0.967	0.940	91.48	0.903	0.846	0.933	0.893	0.852	0.873	0.909	0.269	0.170	0.889	0.054
M10-3	0.960	0.938	76.23	0.837	0.707	0.873	0.827	0.761	0.794	0.848	0.349	0.205	0.726	0.117
M10-4	0.959	0.934	74.54	0.839	0.709	0.877	0.828	0.763	0.796	0.849	0.348	0.203	0.737	0.112
M10-5	0.957	0.933	70.15	0.851	0.776	0.865	0.838	0.776	0.808	0.866	0.342	0.189	0.747	0.114

Table S4. Statistical validation of the models predicting antitrypanosomal activity.

Model	R^2	Q^2	F	R_0^2	$R_0'^2$	R_p^2	Q_{F1}^2	Q^2_{F2}	Q^2_{F3}	CCC	MAE	σ_{MAE}	$\overline{R_m^2}$	ΔR_m^2
M11-1	0.948	0.934	133.161	−8.230	0.207	0.329	−9.322	−9.350	−11.153	0.275	1.265	2.967	0.070	0.406
M11-2	0.946	0.932	129.107	−9.310	0.193	0.328	−10.972	−11.004	−13.095	0.259	1.394	3.179	0.070	0.405
M11-3	0.946	0.930	129.600	−8.013	0.219	0.358	−9.130	−9.157	−10.926	0.288	1.271	2.930	0.079	0.423
M11-4	0.942	0.926	118.400	−7.889	0.222	0.362	−8.852	−8.878	−10.599	0.292	1.332	2.849	0.081	0.425
M11-5	0.941	0.926	116.940	−8.383	0.209	0.341	−9.689	−9.718	−11.585	0.276	1.264	3.030	0.073	0.414
M12-1	0.933	0.908	72.703	0.967	0.961	0.971	0.963	0.963	0.957	0.980	0.160	0.093	0.858	0.028
M12-2	0.925	0.905	64.878	0.959	0.951	0.964	0.952	0.952	0.944	0.974	0.189	0.092	0.808	0.040
M12-3	0.923	0.898	63.173	0.973	0.968	0.977	0.966	0.966	0.960	0.982	0.160	0.072	0.889	0.021
M12-4	0.928	0.897	67.821	0.984	0.982	0.988	0.977	0.977	0.973	0.988	0.131	0.065	0.942	0.010
M12-5	0.928	0.897	67.821	0.984	0.982	0.988	0.977	0.977	0.973	0.988	0.131	0.065	0.942	0.010
M13-1	0.932	0.909	54.502	0.973	0.969	0.977	0.973	0.973	0.968	0.986	0.122	0.103	0.843	0.028
M13-2	0.932	0.909	54.838	0.981	0.978	0.983	0.980	0.980	0.977	0.990	0.111	0.078	0.908	0.015
M13-3	0.941	0.908	63.597	0.985	0.982	0.989	0.983	0.983	0.980	0.991	0.119	0.035	0.974	0.008
M13-4	0.925	0.905	49.256	0.971	0.970	0.971	0.970	0.970	0.964	0.984	0.145	0.084	0.880	0.025
M13-5	0.937	0.904	59.040	0.978	0.974	0.983	0.977	0.977	0.973	0.988	0.131	0.060	0.912	0.015
M14-1	0.928	0.906	94.453	0.892	0.867	0.895	0.892	0.892	0.903	0.940	0.238	0.160	0.810	0.072
M14-2	0.880	0.851	53.813	0.878	0.825	0.891	0.876	0.876	0.889	0.927	0.289	0.092	0.683	0.114
M14-3	0.876	0.845	51.714	0.888	0.821	0.918	0.887	0.887	0.899	0.930	0.227	0.188	0.854	0.059
M14-4	0.888	0.844	57.959	0.892	0.826	0.922	0.863	0.863	0.878	0.919	0.288	0.140	0.735	0.084
M14-5	0.865	0.844	47.085	0.852	0.794	0.859	0.851	0.851	0.867	0.914	0.291	0.167	0.598	0.159
M15-1	0.904	0.854	49.292	0.886	0.842	0.896	0.876	0.876	0.889	0.929	0.261	0.161	0.748	0.090
M15-2	0.854	0.810	30.784	0.902	0.839	0.939	0.898	0.898	0.909	0.938	0.229	0.157	0.852	0.048
M15-3	0.868	0.804	34.516	0.673	0.607	0.680	0.673	0.673	0.708	0.820	0.436	0.236	0.285	0.381
M15-4	0.857	0.803	31.539	0.857	0.809	0.862	0.857	0.857	0.872	0.918	0.296	0.139	0.602	0.157
M15-5	0.871	0.800	35.314	0.842	0.750	0.860	0.838	0.838	0.855	0.900	0.317	0.143	0.726	0.109
M16-1	0.935	0.896	57.484	0.976	0.971	0.982	0.974	0.974	0.977	0.986	0.118	0.076	0.971	0.013
M16-2	0.933	0.893	55.710	0.978	0.974	0.984	0.976	0.976	0.979	0.987	0.120	0.058	0.954	0.012

**Model**	R^2	Q^2	F	R_0^2	$R_0'^2$	R_p^2	Q_{F1}^2	${Q^2}_{F2}$	${Q^2}_{F3}$	CCC	MAE	σ_{MAE}	$\overline{R_m^2}$	ΔR_m^2
M16-3	0.937	0.893	59.083	0.884	0.813	0.913	0.884	0.884	0.896	0.928	0.278	0.093	0.744	0.085
M16-4	0.918	0.879	44.655	0.823	0.729	0.836	0.823	0.823	0.842	0.892	0.320	0.176	0.716	0.120
M16-5	0.919	0.874	45.255	0.934	0.906	0.955	0.933	0.933	0.940	0.961	0.212	0.067	0.939	0.015
M17-1	0.861	0.823	45.426	0.938	0.919	0.948	0.921	0.906	0.850	0.946	0.290	0.135	0.859	0.045
M17-2	0.859	0.805	44.764	0.951	0.936	0.962	0.946	0.936	0.897	0.963	0.226	0.140	0.944	0.028
M17-3	0.857	0.803	43.982	0.922	0.887	0.942	0.912	0.896	0.833	0.937	0.277	0.200	0.782	0.065
M17-4	0.846	0.803	40.240	0.923	0.884	0.950	0.920	0.905	0.848	0.942	0.283	0.155	0.870	0.040
M17-5	0.835	0.801	37.122	0.951	0.937	0.960	0.938	0.926	0.882	0.958	0.228	0.174	0.917	0.031
M18-1	0.949	0.915	98.174	0.950	0.936	0.960	0.923	0.909	0.854	0.948	0.252	0.196	0.792	0.049
M18-2	0.914	0.879	55.814	0.985	0.983	0.987	0.966	0.960	0.936	0.978	0.193	0.082	0.949	0.011
M18-3	0.913	0.877	54.931	0.971	0.963	0.982	0.958	0.950	0.920	0.971	0.191	0.139	0.966	0.013
M18-4	0.915	0.875	56.555	0.986	0.985	0.988	0.969	0.963	0.941	0.980	0.181	0.086	0.980	0.009
M18-5	0.914	0.874	55.510	0.985	0.984	0.987	0.967	0.961	0.937	0.979	0.190	0.080	0.920	0.013
M19-1	0.943	0.892	65.608	0.985	0.983	0.987	0.978	0.974	0.958	0.986	0.147	0.087	0.924	0.013
M19-2	0.901	0.863	36.526	0.950	0.938	0.957	0.906	0.889	0.822	0.937	0.303	0.175	0.939	0.032
M19-3	0.922	0.856	47.493	0.953	0.940	0.963	0.945	0.934	0.895	0.963	0.215	0.165	0.894	0.032
M19-4	0.908	0.854	39.382	0.965	0.964	0.965	0.944	0.934	0.895	0.966	0.242	0.117	0.910	0.028
M19-5	0.911	0.850	40.742	0.938	0.942	0.942	0.933	0.921	0.873	0.961	0.216	0.209	0.687	0.100

Table S5. Molecular descriptors used in quantitative structure-activity relationships (QSAR) modeling.

Molecular descriptor	Description
AM1_dipole	Dipole moment
AM1_E	The total SCF energy (kcal/mol)
AM1_Eele	The electronic energy (kcal/mol)
AM1_HF	The heat of formation (kcal/mol)
AM1_HOMO	HOMO energy (eV)
AM1_IP	The ionization potential (kcal/mol)
AM1_LUMO	LUMO energy (eV)
apol	Sum of the atomic polarizabilities
ASA	Water accessible surface area
ASA+	Positive accessible surface area
ASA-	Negative accessible surface area
ASA_H	Water accessible surface area of all hydrophobic atoms
ASA_P	Water accessible surface area of all polar atoms
a_acc	Number of hydrogen bond acceptor atoms (not counting acidic atoms but counting atoms that are both hydrogen bond donors and acceptors such as -OH)
a_count	Number of atoms (including implicit hydrogens)
a_don	Number of hydrogen bond donor atoms (not counting basic atoms but counting atoms that are both hydrogen bond donors and acceptors such as -OH)
a_donacc	Number of hydrogen bond donor plus number of hydrogen bond acceptor atoms
a_heavy	Number of heavy atoms
a_hyd	Number of hydrophobic atoms
a_IC	Atom information content (total)
a_ICM	Atom information content (mean)
a_nC	Number of carbon atoms
a_nH	Number of hydrogen atoms
a_nO	Number of oxygen atoms
balabanJ	Balaban's connectivity topological index
BCUT_PEOE_0	Smallest BCUT descriptor using PEOE partial charges
BCUT_PEOE_1	Second BCUT descriptor using PEOE partial charges
BCUT_PEOE_2	Third BCUT descriptor using PEOE partial charges
BCUT_PEOE_3	Largest BCUT descriptor using PEOE partial charges
BCUT_SLOGP_0	Smallest BCUT descriptor using atomic contribution to logP
BCUT_SLOGP_1	Second BCUT descriptor using atomic contribution to logP
BCUT_SLOGP_2	Third BCUT descriptor using atomic contribution to logP
BCUT_SLOGP_3	Largest BCUT descriptor using atomic contribution to logP
BCUT_SMR_0	Smallest BCUT descriptor using atomic contribution to molar refractivity
BCUT_SMR_1	Second BCUT descriptor using atomic contribution to molar refractivity
BCUT_SMR_2	Third BCUT descriptor using atomic contribution to molar refractivity
BCUT_SMR_3	Largest BCUT descriptor using atomic contribution to molar refractivity

Molecular descriptor	Description
bpol	Sum of the absolute value of the difference between atomic polarizabilities of all bonded atoms in the molecule
b_1rotN	Number of rotatable single bonds
b_1rotR	Fraction of rotatable single bonds
b_count	Number of bonds (including implicit hydrogens)
b_heavy	Number of bonds between heavy atoms
b_max1len	Length of the longest single bond chain
b_rotN	Number of rotatable bonds
b_rotR	Fraction of rotatable bonds
b_single	Number of single bonds
CASA+	Charge-weighted positive surface area
CASA−	Charge-weighted negative surface area
chi0	Atomic connectivity index (order 0)
chi0v	Atomic valence connectivity index (order 0)
chi0v_C	Carbon valence connectivity index (order 0)
chi0_C	Carbon connectivity index (order 0)
chi1	Atomic connectivity index (order 1)
chi1v	Atomic valence connectivity index (order 1)
chi1v_C	Carbon valence connectivity index (order 1)
chi1_C	Carbon connectivity index (order 1)
DASA	Absolute difference in surface area
DCASA	Absolute difference in charge-weighted areas
dens	Mass density (AMU/A^3)
density	Molecular mass density
diameter	Largest value in the distance matrix
dipole	Dipole moment
dipoleX	The x component of the dipole moment
dipoleY	The y component of the dipole moment
dipoleZ	The z component of the dipole moment
E	Value of the potential energy
E_ang	Angle bend potential energy
E_ele	Electrostatic component of the potential energy
E_nb	Value of the potential energy with all bonded terms disabled
E_sol	Solvation energy
E_str	Bond stretch potential energy
E_strain	Local strain energy: the current energy minus the value of the energy at a near local minimum
E_tor	Torsion (proper and improper) potential energy
E_vdw	Van der Waals component of the potential energy
FASA+	Fractional positive accessible surface area
FASA−	Fractional negative accessible surface area
FASA_H	Fractional hydrophobic surface area
FASA_P	Fractional polar surface area
FCASA+	Fractional charge-weighted positive surface area

Molecular descriptor	Description
FCASA–	Fractional charge-weighted negative surface area
GCUT_PEOE_0	Smallest GCUT descriptor using PEOE partial charges
GCUT_PEOE_1	Second GCUT descriptor using PEOE partial charges
GCUT_PEOE_2	Third GCUT descriptor using PEOE partial charges
GCUT_PEOE_3	Largest GCUT descriptor using PEOE partial charges
GCUT_SLOGP_1	Second GCUT descriptor using atomic contribution to logP
GCUT_SLOGP_2	Third GCUT descriptor using atomic contribution to logP
GCUT_SLOGP_3	Largest GCUT descriptor using atomic contribution to logP
GCUT_SMR_0	Smallest GCUT descriptor using atomic contribution to molar refractivity
GCUT_SMR_1	Second GCUT descriptor using atomic contribution to molar refractivity
GCUT_SMR_2	Third GCUT descriptor using atomic contribution to molar refractivity
GCUT_SMR_3	Largest GCUT descriptor using atomic contribution to molar refractivity
glob	Globularity
h_ema	Sum of hydrogen bond acceptor strengths
h_emd	Sum of hydrogen bond donor strengths
h_emd_C	Sum of hydrogen bond donor strengths of carbon atoms
h_logD	The octanol/water distribution coefficient at pH 7
h_logP	Log of the octanol/water partition coefficient using an 8 parameter model based on Hueckel Theory
h_logS	Log of the aqueous solubility (mol/L) using a 7 parameter model based on Hueckel Theory
h_log_pbo	Sum of log (1 + pi bond order) for all bonds
h_mr	Molar refractivity using a 4 parameter model based on Hueckel Theory
h_pavgQ	The average total charge
h_pKa	The pKa of the reaction that removes a proton from the ensemble of states with a hydrogen count equal to the input structure
h_pKb	The pKb of the reaction that adds a proton from the ensemble of states with a hydrogen count equal to the input structure
h_pstates	The entropic count or fractional number of protonation states
h_pstrain	The strain energy (kcal/mol) needed to convert all protonation states into the input protonation state
Kier1	First kappa shape index
Kier2	Second kappa shape index
Kier3	Third kappa shape index
KierA1	First alpha modified shape index
KierA2	Second alpha modified shape index
KierA3	Third alpha modified shape index
KierFlex	Kier molecular flexibility index
lip_acc	The number of O and N atoms
lip_don	The number of OH and NH atoms
logP(o/w)	Log octanol/water partition coefficient
logS	Log of the aqueous solubility (mol/L)
MNDO_dipole	Dipole moment
MNDO_E	The total SCF energy (kcal/mol)
MNDO_Eele	The electronic energy (kcal/mol)

Molecular descriptor	Description
MNDO_HF	The heat of formation (kcal/mol)
MNDO_HOMO	HOMO energy (eV)
MNDO_IP	The ionization potential (kcal/mol)
MNDO_LUMO	LUMO energy (eV)
mr	Molecular refractivity
npr1	Normalized PMI ratio (1) (pmi1 / pmi3)
npr2	Normalized PMI ratio (2) (pmi2 / pmi3)
opr_nrot	The number of rotatable bonds
PEOE_PC+	Total positive partial charge using the Partial Equalization of Orbital Electronegativities (PEOE) method
PEOE_PC–	Total negative partial charge using PEOE method
PEOE_RPC+	Relative positive partial charge using PEOE method
PEOE_RPC–	Relative negative partial charge using PEOE method
PEOE_VSA+0	Sum of van der Waals surface area where partial charge is in the range (0.00,0.05)
PEOE_VSA+1	Sum of van der Waals surface area where partial charge is in the range (0.05,0.10)
PEOE_VSA+2	Sum of van der Waals surface area where partial charge is in the range (0.10,0.15)
PEOE_VSA+4	Sum of van der Waals surface area where partial charge is in the range (0.20,0.25)
PEOE_VSA-0	Sum of van der Waals surface area where partial charge is in the range (–0.05,0.00)
PEOE_VSA-1	Sum of van der Waals surface area where partial charge is in the range (–0.10,–0.05)
PEOE_VSA-6	Sum of van der Waals surface area where partial charge is less than –0.30
PEOE_VSA_FHYD	Fractional hydrophobic van der Waals surface area
PEOE_VSA_FNEG	Fractional negative van der Waals surface area
PEOE_VSA_FPNEG	Fractional negative polar van der Waals surface area
PEOE_VSA_FPOL	Fractional polar van der Waals surface area
PEOE_VSA_FPOS	Fractional positive van der Waals surface area
PEOE_VSA_FPPOS	Fractional positive polar van der Waals surface area
PEOE_VSA_HYD	Total hydrophobic van der Waals surface area
PEOE_VSA_NEG	Total negative van der Waals surface area
PEOE_VSA_PNEG	Total negative polar van der Waals surface area
PEOE_VSA_POL	Total polar van der Waals surface area
PEOE_VSA_POS	Total positive van der Waals surface area
PEOE_VSA_PPOS	Total positive polar van der Waals surface area
PM3_dipole	Dipole moment
PM3_E	The total SCF energy (kcal/mol)
PM3_Eele	The electronic energy (kcal/mol)
PM3_HF	The heat of formation (kcal/mol)
PM3_HOMO	HOMO energy (eV)
PM3_IP	The ionization potential (kcal/mol)
PM3_LUMO	LUMO energy (eV)

Molecular descriptor	Description
pmi	Principal moment of inertia
pmi1	First diagonal element of diagonalized moment of inertia tensor
pmi2	Second diagonal element of diagonalized moment of inertia tensor
pmi3	Third diagonal element of diagonalized moment of inertia tensor
pmiX	x component of the principal moment of inertia
pmiY	y component of the principal moment of inertia
pmiZ	z component of the principal moment of inertia
Q_PC+	Total positive partial charge using partial charges in the input structure
Q_PC-	Total negative partial charge
Q_RPC+	Relative positive partial charge
Q_RPC-	Relative negative partial charge
Q_VSA_FHYD	Fractional hydrophobic van der Waals surface area
Q_VSA_FNEG	Fractional negative van der Waals surface area
Q_VSA_FPNEG	Fractional negative polar van der Waals surface area
Q_VSA_FPOL	Fractional polar van der Waals surface area
Q_VSA_FPOS	Fractional positive van der Waals surface area
Q_VSA_FPPOS	Fractional positive polar van der Waals surface area
Q_VSA_HYD	Total hydrophobic van der Waals surface area
Q_VSA_NEG	Total negative van der Waals surface area
Q_VSA_PNEG	Total negative polar van der Waals surface area
Q_VSA_POL	Total polar van der Waals surface area
Q_VSA_POS	Total positive van der Waals surface area
Q_VSA_PPOS	Total positive polar van der Waals surface area
radius	Smallest entry in the distance matrix
rgyr	Radius of gyration
rsynth	synthetic reasonableness, or feasibility, of the chemical structure
SlogP	Log of the octanol/water partition coefficient
SlogP_VSA0	Sum of van der Waals surface area where the contribution to SlogP is ≤ -0.4
SlogP_VSA3	Sum of van der Waals surface area where the contribution to SlogP is in the range (0,0.1)
SlogP_VSA8	Sum of van der Waals surface area where the contribution to SlogP is in the range (0.30,0.40)
SlogP_VSA9	Sum of van der Waals surface area where the contribution to SlogP is > 0.40
SMR	Molecular refractivity
SMR_VSA1	Sum of van der Waals surface area where the contribution to SMR is in the range (0.11,0.26)
SMR_VSA3	Sum of van der Waals surface area where the contribution to SMR is in the range (0.35,0.39)
SMR_VSA5	Sum of van der Waals surface area where the contribution to SMR is in the range (0.44,0.485)
SMR_VSA6	Sum of van der Waals surface area where the contribution to SMR is in the range (0.485,0.56)
SMR_VSA7	Sum of van der Waals surface area where the contribution to SMR is > 0.56
std_dim1	Standard dimension one
std_dim2	Standard dimension two

Molecular descriptor	Description
std_dim3	Standard dimension three
TPSA	Polar surface area (Å2)
VAdjEq	Vertex adjacency information (equality)
VAdjMa	Vertex adjacency information (magnitude)
VDistEq	If *m* is the sum of the distance matrix entries then VdistEq is defined to be the sum of log2 *m* - *pi* log2 *pi* / m where pi is the number of distance matrix entries equal to i.
VDistMa	If *m* is the sum of the distance matrix entries then VDistMa is defined to be the sum of log2 *m* - *Dij* log2 *Dij* / *m* over all *i* and *j*
vdw_area	Area of van der Waals surface (Å2)
vdw_vol	van der Waals volume (Å3)
vol	Van der Waals volume
VSA	Van der Waals surface area
vsa_acc	Approximation to the sum of VDW surface areas (Å2) of pure hydrogen bond acceptors
vsa_hyd	Approximation to the sum of VDW surface areas of hydrophobic atoms (Å2)
vsa_pol	Approximation to the sum of VDW surface areas (Å2) of polar atoms
vsurf_A	Amphiphilic moment
vsurf_CP	Critical packing parameter
vsurf_CW1	Capacity factor at –0.2
vsurf_CW2	Capacity factor at –0.5
vsurf_CW3	Capacity factor at –1.0
vsurf_CW4	Capacity factor at –2.0
vsurf_CW5	Capacity factor at –3.0
vsurf_CW6	Capacity factor at –4.0
vsurf_CW7	Capacity factor at –5.0
vsurf_CW8	Capacity factor at –6.0
vsurf_D1	Hydrophobic volume at –0.2
vsurf_D2	Hydrophobic volume at –0.4
vsurf_D3	Hydrophobic volume at –0.6
vsurf_D4	Hydrophobic volume at –0.8
vsurf_D5	Hydrophobic volume at –1.0
vsurf_D6	Hydrophobic volume at –1.2
vsurf_D7	Hydrophobic volume at –1.4
vsurf_D8	Hydrophobic volume at –1.6
vsurf_DD12	vsurf_EDmin1, vsurf_EDmin2 distance
vsurf_DD13	vsurf_EDmin1, vsurf_EDmin3 distance
vsurf_DD23	vsurf_EDmin2, vsurf_EDmin3 distance
vsurf_EDmin1	Lowest hydrophobic energy
vsurf_EDmin2	Second lowest hydrophobic energy
vsurf_EDmin3	Third lowest hydrophobic energy
vsurf_EWmin1	Lowest hydrophilic energy
vsurf_EWmin2	Second lowest hydrophilic energy
vsurf_EWmin3	Third lowest hydrophilic energy
vsurf_G	Surface globularity

Molecular descriptor	Description
vsurf_HB1	H-bond donor capacity at –0.2
vsurf_HB2	H-bond donor capacity at –0.5
vsurf_HB3	H-bond donor capacity at –1.0
vsurf_HB4	H-bond donor capacity at –2.0
vsurf_HB5	H-bond donor capacity at –3.0
vsurf_HB6	H-bond donor capacity at –4.0
vsurf_HB7	H-bond donor capacity at –5.0
vsurf_HB8	H-bond donor capacity at –6.0
vsurf_HL1	First hydrophilic-lipophilic balance
vsurf_HL2	Second hydrophilic-lipophilic balance
vsurf_ID1	Hydrophobic integy moment at –0.2
vsurf_ID2	Hydrophobic integy moment at –0.4
vsurf_ID3	Hydrophobic integy moment at –0.6
vsurf_ID4	Hydrophobic integy moment at –0.8
vsurf_ID5	Hydrophobic integy moment at –1.0
vsurf_ID6	Hydrophobic integy moment at –1.2
vsurf_ID7	Hydrophobic integy moment at –1.4
vsurf_ID8	Hydrophobic integy moment at –1.6
vsurf_IW1	Hydrophilic integy moment at –0.2
vsurf_IW2	Hydrophilic integy moment at –0.5
vsurf_IW3	Hydrophilic integy moment at –1.0
vsurf_IW4	Hydrophilic integy moment at –2.0
vsurf_IW5	Hydrophilic integy moment at –3.0
vsurf_IW6	Hydrophilic integy moment at –4.0
vsurf_IW7	Hydrophilic integy moment at –5.0
vsurf_IW8	Hydrophilic integy moment at –6.0
vsurf_R	Surface rugosity
vsurf_S	Interaction field area
vsurf_V	Interaction field volume
vsurf_W1	Hydrophilic volume at –0.2
vsurf_W2	Hydrophilic volume at –0.5
vsurf_W3	Hydrophilic volume at –1.0
vsurf_W4	Hydrophilic volume at –2.0
vsurf_W5	Hydrophilic volume at –3.0
vsurf_W6	Hydrophilic volume at –4.0
vsurf_W7	Hydrophilic volume at –5.0
vsurf_W8	Hydrophilic volume at –6.0
vsurf_Wp1	Polar volume at –0.2
vsurf_Wp2	Polar volume at –0.5
vsurf_Wp3	Polar volume at –1.0
vsurf_Wp4	Polar volume at –2.0
vsurf_Wp5	Polar volume at –3.0
vsurf_Wp6	Polar volume at –4.0
Weight	Molecular weight (CRC)

Molecular descriptor	Description
wienerPath	Wiener path number
wienerPol	Wiener polarity number
zagreb	Zagreb index

Table S6. Constitution of the test sets for QSAR modeling.

Biological activity	Type of selection	Models	Compounds used as test set	
Antileishmanial	Random 1	M1-1 to M1-5	**20**	**5**
		M2-1 to M2-5	**21**	**2**
		M3-1 to M3-5	**11**	**29**
			**28**	**26**
	Random 2	M4-1 to M4-5	**16**	**15**
		M5-1 to M5-5	**17**	**8**
		M6-1 to M6-5	**12**	**3**
			**24**	**33**
	Kennard-Stone algorithm	M7-1 to M7-5	**4**	**7**
		M8-1 to M8-5	**5**	**13**
		M9-1 to M9-5	**14**	**20**
		M10-1 to M10-5	**16**	**30**
Antitrypanosomal	Random 1	M11-1 to M11-5	**2**	**16**
		M12-1 to M12-5	**5**	**23**
		M13-1 to M13-5	**8**	**26**
			**15**	**33**
	Random 2	M14-1 to M14-5	**1**	**22**
		M15-1 to M15-5	**7**	**24**
		M16-1 to M16-5	**14**	**25**
			**17**	**32**
	Kennard-Stone algorithm	M17-1 to M17-5	**4**	**14**
		M18-1 to M18-5	**5**	**16**
		M19-1 to M19-5	**7**	**20**
			**13**	**30**

References

1. Bernal, F.A.; Kaiser, M.; Wünsch, B.; Schmidt, T.J. Structure – Activity Relationships of Cinnamate Ester Analogues as Potent Antiprotozoal Agents. *ChemMedChem* **2019**, *14*, xxx–xxx. DOI: 10.1002/cmdc.201900544.
2. Roy, K.; Kar, S.; Das, R.N. Statistical Methods in QSAR/QSPR. In *A Primer on QSAR/QSPR Modeling*; Springer, Cham, 2015; pp. 37–59 ISBN 9780128015056.
3. Golbraikh, A.; Tropsha, A. Beware of q^2! *J. Mol. Graph. Model.* **2002**, *20*, 269–276.
4. Roy, P.P.; Paul, S.; Mitra, I.; Roy, K. On two novel parameters for validation of predictive QSAR models. *Molecules* **2009**, *14*, 1660–1701.
5. Roy, K.; Mitra, I.; Kar, S.; Ojha, P.K.; Das, R.N.; Kabir, H. Comparative studies on some metrics for external validation of QSPR models. *J. Chem. Inf. Model.* **2012**, *52*, 396–408.

6. Roy, K.; Chakraborty, P.; Mitra, I.; Ojha, P.K.; Kar, S.; Das, R.N. Some case studies on application of "rm2" metrics for judging quality of quantitative structure-activity relationship predictions: Emphasis on scaling of response data. *J. Comput. Chem.* **2013**, *34*, 1071–1082.
7. Schüürmann, G.; Ebert, R.-U.; Chen, J.; Wang, B.; Kühne, R. External Validation and Prediction Employing the Predictive Squared Correlation Coefficient - Test Set Activity Mean vs Training Set Activity Mean. *J. Chem. Inf. Model.* **2008**, *48*, 2140–2145.
8. Consonni, V.; Ballabio, D.; Todeschini, R. Evaluation of model predictive ability by external validation techniques. *J. Chemom.* **2010**, *24*, 194–201.
9. Chirico, N.; Gramatica, P. Real external predictivity of QSAR models: How to evaluate It? Comparison of different validation criteria and proposal of using the concordance correlation coefficient. *J. Chem. Inf. Model.* **2011**, *51*, 2320–2335.
10. Lin, L.I.-K. A Concordance Correlation Coefficient to Evaluate Reproducibility. *Biometrics* **1989**, *45*, 255–268.
11. Roy, K.; Das, R.N.; Ambure, P.; Aher, R.B. Be aware of error measures. Further studies on validation of predictive QSAR models. *Chemom. Intell. Lab. Syst.* **2016**, *152*, 18–33.
